# Economic, social, and clinic influences on opioid treatment program retention in Dar es Salaam, Tanzania: a qualitative study

**DOI:** 10.1186/s13722-023-00374-1

**Published:** 2023-03-27

**Authors:** Deja Knight, Iddi Haruna Nkya, Nora Solon West, Cui Yang, Michael Kidorf, Carl Latkin, Haneefa T. Saleem

**Affiliations:** 1 Department of International Health, Bloomberg School of Public Health, Johns Hopkins University, 615 North Wolfe Street, Baltimore, MD 21205 USA; 2 Department of Psychiatry and Mental Health, School of Medicine, Muhimbili University of Health and Allied Sciences, P.O. Box 65001, Dar Es Salaam, Tanzania; 3 Department of Health, Behavior, and Society, Bloomberg School of Public Health, Johns Hopkins University, 2213 McElderry Street, 2nd Floor, Baltimore, MD 21205 USA; 4 Department of Psychiatry and Behavioral Sciences, School of Medicine, Johns Hopkins University, Johns Hopkins Bayview Medical Campus, 5510 Nathan Shock Drive, Baltimore, MD 21224 USA; 5 Department of Health, Behavior, and Society, Bloomberg School of Public Health, Johns Hopkins University, 624 North Broadway Avenue, Hampton House Room 737, Baltimore, MD 21205 USA

**Keywords:** Medications for opioid use disorder, Opioid treatment, Retention, Qualitative, Tanzania

## Abstract

**Background:**

Medications for opioid use disorder (MOUD) are associated with positive health outcomes. People remaining on MOUD have a reduced likelihood of drug overdose and mortality. Tanzania supports a national opioid treatment program (OTP) offering MOUD, but retention is a continual challenge. To date, most research on MOUD retention in Tanzania and other Sub-Saharan Africa settings has been focused on the individual-level, with little attention to economic, social, and clinic-level factors.

**Methods:**

We qualitatively examined economic, social, and clinic factors that affect retention on MOUD, specifically methadone maintenance therapy, among former and current clients attending an OTP clinic Dar es Salaam, Tanzania. We conducted in-depth interviews with a total of 40 current and former clients receiving MOUD and four focus groups with an additional 35 current clients on MOUD between January and April 2020. We utilized a thematic analysis approach.

**Results:**

Daily OTP clinic attendance posed a financial burden to current and former clients and was a barrier to remaining on MOUD. Though treatment is free, clients described struggles to attend clinic, including being able to afford transportation. Female clients were differentially impacted, as sex work was the most common income-generating activity that they participated in, which presented its own set of unique challenges, including barriers to attending during set clinic hours. Drug use stigma acted as a barrier to MOUD and prevented clients from securing a job, rebuilding trust within the community, and accessing transportation to attend the clinic. Being able to rebuild trust with family facilitated remaining on MOUD, as family provided social and financial support. Caretaking responsibilities and familial expectations among female clients conflicted with MOUD adherence. Finally, clinic level factors, such as clinic dispensing hours and punitive consequences for breaking rules, posed barriers to clients on MOUD.

**Conclusion:**

Social and structural factors, both within (e.g., clinic policies) and outside of (e.g., transportation) the clinic impact MOUD retention. Our findings can inform interventions and policies to address economic and social barriers to MOUD, that can contribute to sustained recovery.

## Introduction

Medications for opioid use disorder (MOUD) are associated with positive health outcomes. People on MOUD have lower HIV and HCV risks [1, 2], reduced rates of overdose and mortality [3], greater access to other medical services [4], and greater likelihood of other positive treatment outcomes, such as improvements in physical and mental health, HIV viral suppression, reduced heroin use, fewer legal problems and less criminal involvement, and improved maternal and fetal outcomes among pregnant women [5–7]. Discontinuing MOUD against medical advice is associated with increased drug use, including injection drug use, other risky behaviors, legal problems, community harms, overdose risks, mortality, and disengagement from other treatment, such as HIV and mental health treatment [3, 8].

In Tanzania, a publicly funded opioid treatment program (OTP) was established in 2011 at the Muhimbili National Hospital (MNH) in Dar es Salaam. There are currently 12 OTP clinics throughout the country, including three in Dar es Salaam. The OTP offers MOUD, specifically methadone maintenance therapy, as well as an array of other pharmacological, behavioral, and psychosocial support services, such as tuberculosis testing and treatment, clinical medical services, psychotherapy, and case management through on-site social workers at no cost. Community-based organizations (CBOs) provide harm-reduction services and link people who use drugs to OTP clinics. Facility-based and CBO-based outreach workers are also involved in providing support for enrollment and retention. Despite the broad range of free services available to address the multiple needs of clients, retention remains a challenge. [9]. There is variability in client retention rates across OTP clinics. At the MNH OTP clinic, only 64% of individuals receiving MOUD are retained in treatment at 12 months compared to 73% at another OTP clinic in Dar es Salaam [10], suggesting room for improvement.

To date, published research on MOUD retention in Tanzania has been mainly quantitative and focused on individual factors, such as demographic factors, like age, employment status, and sex, methadone dose, injecting behaviors, and history of abuse as predictors of attrition [9, 10]. And though travel distance to the clinic has been shown to affect MOUD retention [10], there has been little elaboration on the mechanisms through which economic, clinic, and social factors affect MOUD retention. Economic barriers to MOUD, such as treatment fees and transportation costs, have been documented in other settings [11, 12]. While the MOUD services in Tanzania are free to clients, other costs, such as transportation, may pose a challenge for clients attending clinic daily. Research examining the effects of indirect treatment costs on MOUD retention in Tanzania is limited. Clinic factors, such as treatment length, dosing policy, the clinic environment, including staff, waiting times, and supportive services, have been shown to affect retention on MOUD [13, 14]. In Tanzania, as in many other settings, social relationships are often critical to health seeking behaviors and health outcomes of people who use drugs [15, 16]. Interdependence and collectivism are important in mobilizing resources to seek care, such as transportation and childcare to attend clinic appointments. Yet there is little information in this context about how social relationships influence MOUD retention and treatment success. Relationships with OTP staff and other clinic-level elements, may also play a role in treatment retention [12] but have not been fully explored in this setting in the context of treatment retention.

In this paper we qualitatively examine economic, social, and clinic factors that affect MOUD retention among former and current clients at an OTP clinic in Dar es Salaam, Tanzania.

## Methods

### Study setting

The study was conducted at the MNH OTP clinic in Dar es Salaam, Tanzania. Clients on MOUD attend clinic daily to receive their methadone medication, unless they are among the few clients who meet the criteria for takeaway doses. Methadone is dispensed at the pharmacy window between 4 and 9 a.m.—clients with scheduled appointments with a physician or social worker can receive methadone up until 12 p.m.—and consumption is directly observed by the pharmacist or pharmacist technician. Clients also meet with physicians at set intervals: enrollment, three days after enrollment, and at least once a week until treatment stabilization is reached. Once the methadone dose is stabilized for a client, they meet with the physician every two to three months. In addition to meeting with physicians, clients undergo random urinalysis testing for multiple substances, including heroin. Clients who test positive for heroin on a urine drug screen are considered for adjustments to their methadone dose. For clients who test positive for cannabis or benzodiazepines, their methadone dose is reviewed for adjustments, and they are referred to a social worker at the clinic for additional counseling and required to attend additional educational sessions at affiliated CBOs. Methadone is not dispensed to clients who are found to have high blood alcohol content in breath samples tested using a breathalyzer. Mutual 12-step support group meetings, such as Alcoholics Anonymous (AA) and Methadone Anonymous (MA), are offered through the clinic and the four CBOs that provide community-based harm reduction services for people who use drugs and link individuals to the OTP clinic.

### Study sample and procedures

We conducted qualitative in-depth interviews and focus group discussions with current and former clients receiving MOUD between January and April 2020, prior to the COVID-19 pandemic. Current clients on MOUD were recruited through the Muhimbili OTP clinic and former clients were recruited through community outreach workers who work at the CBOs. We purposively sampled study participants based on MOUD enrollment status and gender.

We conducted in-depth interviews with a total of 40 current and former MOUD clients. We used the Berkman et al. conceptual model on how social networks affect health to guide the development of the semi-structured interview guide [17]. The Berkman model represents social networks as embedded in a larger structural context in which structural factors affect how social networks are formed and sustained. Social networks are posited as operating at the behavioral level through four primary processes: (1) social support, (2) social influence, (3) social engagement and attachment, and (4) access to resources. These processes then influence more proximate pathways to health, such as health-seeking behaviors. The interview guide included broad questions on barriers and facilitators to MOUD retention as well as more focused questions on social support, positive and negative social influences, social engagement, and access to resources, including financial resources, that support MOUD retention, in line with the Berkman model. Interviews were conducted one-on-one with trained interviewers in Swahili in private rooms at the study office. Interviews lasted approximately 45 min to 1 h and were audio-recorded, transcribed in Swahili, and translated to English.

We conducted four focus groups with 35 current clients. To explore gender differences in MOUD experiences, focus groups were stratified by gender, with two focus groups with men (n = 20) and two with women (n = 15). Trained facilitators led each focus group and used a discussion guide that focused on barriers and facilitators to MOUD enrollment and retention with additional probing questions on economic barriers to MOUD retention, short- and long-term goals, and income-generation. Focus groups lasted approximately 1–1.5 h and were audio-recorded, transcribed in Swahili, and translated to English. All participants provided informed consent.

### Analytic approach

Data analysis followed an iterative process. The study team met weekly during the data collection period to discuss emergent cross-cutting themes, refine study instruments, and address any logistical challenges in data collection. We developed a codebook that contained both deductive and inductive codes. Deductive codes were derived from the Berkman model and study objectives. Inductive codes were identified through the reading of the transcripts and team discussion and drew from grounded theory coding procedures [18]. To generate inductive codes, the last author read through a sample of interview transcripts and conducted line-by-line coding. Line-by-line codes were then synthesized into focused codes, which represented recurrent patterns in the data. We used a constant comparative method to compare codes and coding within and across transcripts, and to refine inductive codes. We used the final codebook with deductive and inductive codes to code all transcripts. Following coding, we explored quotes for each code and compared the relationship between codes to identify common themes. For example, codes related to transportation and income generation informed interpretations of economic influences on MOUD retention for clients. We also generated matrices and memos to organize and examine the information for patterns and to capture connections and comparisons. Data management and coding were conducted using NVivo (QSR International, Melbourne, Australia).

### Ethical considerations

The study was approved by ethical review committees at the Muhimbili University of Health and Allied Sciences, the National Institute for Medical Research in Tanzania, and the Johns Hopkins University Bloomberg School of Public Health. We obtained permission to conduct the study from the Muhimbili National Hospital. All study participants provided informed consent prior to participation. To maintain confidentiality names and other identifying information have been omitted.

## Results

### Participant characteristics

Interview participant characteristics are summarized in Table 1. Interview participants were majority male (57.5%) with a median age of 40.5 years. Most interview participants were current MOUD clients (72.5%). Overall, the median time enrolled on MOUD was 36 months.

**Table 1:** Interview participant characteristics (n = 40)

Interview characteristics	
Age (median)	40.5 years (Range: 21 to 56 years)
Sex	
Women	17
Men	23
MOUD enrollment status	
Current client	29
Former client	11
Time since enrollment on MOUD (median)	3 years (Range: < 1 year – 17 years)
Education level	
Primary or less	16
Secondary or higher	5
Unspecified	19
Partner drug use status	
Current MOUD client	10
Former MOUD client	1
Currently uses drugs	1
No history of drug use	18
No partner	10

### Key influences on MOUD retention

Economic influences, drug use stigma, social support, and clinic policies emerged as key overarching factors that affected the ability of clients to remain on treatment. These factors were interrelated and combined to create compounded barriers to or support for MOUD retention. Economic influences included transportation costs and time conflicts with work schedules. Drug use stigma underlined many of the barriers encountered by clients and manifested as economic challenges due to limited income-generation opportunities and conflicts with families that limited social support. Social support encompassed several themes including reconciliation with family, trust, and access to resources, which frequently overlapped with economic influences. Clinic policies, specifically methadone dosing daily schedules and policies around violations of clinic rules, at times intersected with themes around transportation and work schedule conflicts. Each of these influences on MOUD retention is further explored below.

#### Economic influences on MOUD retention

Daily OTP clinic attendance was reported as a financial burden to current and former clients on MOUD due to transportation costs, particularly bus fare:

“I woke up and I had no money to board a bus...no money to take a motorcycle and…I went to some shops to borrow money…they refused…So the time that you have been trying to borrow money, you waste a lot of time. So, I decide to not attend at the clinic…I definitely see that income is one of the reasons that makes me fail to attend the clinic most of the time.” (31-year-old current female client)

Therefore, being able to earn income or receiving financial support from others was described as an important facilitator for remaining on MOUD.

Though income-generating activities and other financial support were perceived as critical to overcoming the financial burden of attending the clinic, many former clients described the opportunity costs as a barrier to MOUD retention for clients employed:

“I told [OTP clinic staff] that I am defaulting [from the OTP] because the schedule of going to the clinic and back home every day is interfering with my work schedule.” (45-year-old former male client)

Gender inequities exacerbated the economic vulnerability of women receiving MOUD. Many participants, particularly female participants, reported how sex work is often one of the few options for female clients to earn money. Sex work was described by participants as placing women at higher risk of defaulting or being suspended from receiving MOUD. Sex work hours and substance use associated with sex work presented unique barriers to MOUD retention for women. As one female participant in a focus group explained:

“I go to the entertainment places [popular for finding sex clients] to look for money, until at 5 am or 6 am. I am so tired because when you are going to the road [going to sell sex], you can never do it without alcohol. And so, you will say, let me drink at least 1 to 2 bottles of alcohol so that I can have enough “steam” to go to the road [sell sex]. After returning from the road, you are so tired and sleep. You decide to force yourself to take shower and sleep a little bit. When you wake up, it is already 8 am and the [OTP clinic] services end at 9 a.m. When you arrive here at the clinic, it is already late and you will miss the dose, which means you will be in so much pain…that is when you will find people returning to using heroin.” (Women-Only Focus Group 2)

Though sex work allowed some female clients to overcome economic barriers to remaining on MOUD, clinic factors exacerbated challenges for female clients. Peak sex work hours and the nature of sex work conflicted with early morning clinic pharmacy dispensing hours. Clinic policy to deny methadone dispensing to any client with a positive alcohol test was a related obstacle to treatment adherence and retention.

#### Drug use stigma as a barrier to remaining on MOUD

Drug use stigma was reported by all participants, regardless of gender or MOUD enrollment status, as a significant barrier to remaining on MOUD, and at times overlapped with economic barriers. Some participants reported not being able to secure jobs because of their history of drug use and mistrust that they continued to face in the community, which prevented them from earning income that could help support MOUD retention. Some participants described public bus drivers not stopping for them or forcing them off the bus early because of their history of drug use and past behaviors of stealing. These challenges often led clients to arrive late at the clinic or to miss their daily methadone dose completely. Repeated challenges attending clinic was portrayed as leading some clients to default from treatment (defined as having missed 30 consecutives daily methadone doses).

#### Rebuilding trust with family and social support as facilitators of MOUD retention

Throughout interviews and focus groups, participants described how enrollment in the OTP was often a means for clients to rebuild trust with family. Many participants described fractured relationships with family and others in the broader community, and isolation from family, prior to taking MOUD.

“In the past when I was addicted to drugs, I was living in a rough environment because of how I was getting money to go and buy drugs. We would even sleep with a man even if it was unwillingly as long as you get money to buy drugs. Another challenge was selling your clothes and you must beg relatives to give you some. It reaches the point where those relatives get fed up with you and isolate themselves from you because you do not change. But now ever since I stopped using drugs [after taking MOUD], I have my family back and they love me.” (41-year-old former female client)

Restored relationships with family and intimate partner relationships were described by participants as important sources of social support, particularly financial and other instrumental support, that help to overcome economic barriers to MOUD retention. Social support from family and partners often took the form of financial support such as bus fare to attend the clinic daily, food, housing, and emotional support including  encouragement to remain in treatment. Regained respect and acceptance from family motivated some participants to remain in treatment:

“Drugs put me in a bad situation because I was alienated by the community, family, and friends. Brothers, relatives, and friends had all isolated me. Honestly, after joining methadone treatment, slowly people have started to accept me…My daughter, my parent, my closest people, and friends have returned love. It’s something that gives me so much support that I said that I should just continue to use methadone because I returned to a community that was alienating me, but now I see that the community has started slowly to accept me.” (44-year-old current male client)

Though support from family was seen as a major facilitator of MOUD retention, a few participants reported that clients financially supported by family while receiving MOUD at the OTP clinic risked having family discontinue their financial support due to misunderstandings of treatment duration:

“Family members get tired of you and that you have been attending the clinic for almost 3 to 4 years now and you have not completed your [methadone] dose. So, they [family members] start questioning you. They only see that you are always attending at the treatment. There is other [family members] who expect that you would take the [methadone] dose for 3 to 4 months and then you would finish.” (51-year-old former male client)

Some participants also said methadone side effects, such as drowsiness, could be mistaken for drug use and influence continued mistrust and family financial support. For female participants, caretaking and other domestic responsibilities were viewed as conflicting with the time commitment of daily clinic attendance:

“Yeah, the challenge is the time is limited. For example, I have young children at home. Since the school is closed then I must prepare for them, to cook, to prepare their breakfast then to leave. You will find me thinking “here I have to cook tea and to put it in the thermos, to wake up the youngest one and shower her”, then I leave her to go back in bed… At the same time, I look at the time. . . You see it is better if the clinic hours could end at 12 p.m. or 11 a.m.” (36-year-old current female client)

#### Clinic factors that facilitated and hindered MOUD retention

Participants generally reported health care providers at the OTP clinic as being supportive and non-discriminatory. However, many of the former clients interviewed also pointed to certain clinic policies as punitive and a hindrance to treatment: “You know, when you are punished more often, you get tired.” (49-year-old former male client) These clinic policies included being denied methadone after the pharmacy window closes and being temporarily suspended from treatment due to testing positive for alcohol or heroin use. Participants described how many clients view these policies as burdensome and felt these policies were a major reason that some clients discontinue MOUD use:

“Telling a client not to take their dose for almost a week is like giving them a death penalty. The client sees as if you are giving them a death penalty. And when he thinks of the pain that he will experience, that hurts! This person is aware of how withdrawal is and now he is given such a punishment…You are suspending a person for a whole month, or you are giving him 2 weeks, the pain that the client will be experiencing will definitely make him do something that will be out of his control and probably could lead him to drop out of the treatment.” (31-year-old current female client)

Many participants recommended that the clinic staff offer alternative consequences to violating clinic rules that would not inadvertently require them to miss doses, since they believed missing methadone doses might increase the likelihood that a client would default from treatment.

A few participants, mainly current clients, acknowledged that clients are informed of clinic policies and understand the health-related justification for certain clinic policies. They viewed strict clinic rules as necessary and, at times, blamed clients for violating these rules:

“The motive and intension of deciding to join methadone treatment is because we want to stop using illicit drugs. So, if you have decided to join the treatment and later on you step back to engage yourself with illicit drugs again, then it’s a problem… [Strict clinic rules] should continue because they change us to become good people. (Male-Only Focus Group 1)

In these cases, participants often talked about the importance of self-determination and persistence to remain in treatment, despite clinic rules:

“If you are able to win your heart and you are suspended today, but tomorrow you return and you are suspended the next day and still you return, you can remain on the program.” (Male-Only Focus Group 1)

Participants in both interviews and focus groups reported that the OTP clinic pharmacy dispensing hours (4 a.m. to 9 a.m.) were a major barrier to MOUD retention.

## Discussion

### Summary of findings

We qualitatively examined how economic, social, and clinic factors affected MOUD retention from the perspectives of current and former clients at an OTP clinic in Dar es Salaam, Tanzania. Economic influences, drug use stigma and discrimination, social support, and clinic policies emerged as key themes. We found that these factors combine to create compounded barriers or support systems for MOUD. Study participants reported challenges with bus fare to travel to the clinic daily. Those without income generating activities often relied on their family for financial and other instrumental support. However, those supports are often unsustainable given a long-term treatment commitment. Though participants perceived social support from family as critical to retention, they also noted that family misunderstanding of treatment duration and mistrust might result in discontinuing this support or influencing clients to leave treatment prematurely. While some participants reported trying to get a job, drug use stigma often prevented them from successfully being employed. These economic challenges, coupled with stigma, threatened MOUD retention. Female clients faced unique compounding barriers to treatment retention due to engagement in sex work because of gendered inequities in economic opportunities and domestic responsibilities, including expectations of caregiving. Clinic factors, such as policies and operating hours, posed challenges to clients in receiving their daily methadone doses and often overlapped with economic factors. Taken together, economic factors, drug use stigma, social support networks, and clinic policies and procedures were all reported as major factors affecting the ability of clients to remain on MOUD.

### Implications of findings

In the current study, we found economic challenges is a pervasive barrier to MOUD retention and the well-being of clients. Research has shown that clients on MOUD who obtain and maintain employment have better recovery outcomes [19–21] by reducing their risk of relapsing and increasing their ability to abstain from opioid use compared to their unemployed counterparts [19, 22, 23]. According to Magura et al., employment leads to better outcomes through clients having access to a legal and steady source of income, having an activity to structure their time, and through improvement in self-esteem [20]. Additionally, Kidorf et al. found that when an employment requirement was added to an OTP, those clients who failed to find and maintain employment engaged in more illicit drug use compared to those who found employment positions, demonstrating the importance of employment in the recovery process [23]. This literature is consistent with what we saw in our sample of clients on MOUD. Clients who reported an income generating activity had a source of income that could be used to pay for transportation to clinic and they came to the clinic at a consistent time to get to work on time. While the clinic does not help clients to obtain employment directly, they have initiated ways to train clients on job-related skills. Clients can learn ways to earn income through an occupational therapy and Income generating activities program offered through the OTP. However, in the future, collaborations between the OTP and other sectors to provide additional job training workshops or employment opportunities may be of benefit.

Stigma was also identified as a major barrier to MOUD retention. The role of stigma in MOUD retention has been well documented [24, 25]. People who face drug use stigma are shown to have poorer health outcomes compared to those who do not report stigma [26]. In the present study, clients attempted to overcome these challenges by rebuilding trust with family and the community after enrolling to receive MOUD. However, this was an ongoing process. Our findings highlight the need to address interpersonal and community-level drug use stigma throughout recovery, which could improve treatment retention and long-term recovery. Few interventions have been evaluated for their effectiveness in reducing drug use stigma, and even fewer across diverse cultural settings and populations [27]. Interventions focused on communicating positive depictions of people with substance use disorder and those integrating motivational interviewing have been found to decrease stigmatizing attitudes toward people who use heroin and people with alcohol use disorder, respectively [28, 29]. Prior studies attempting to reduce stigma in other stigmatized areas (e.g., HIV) have used culturally tailored messaging in mass media campaigns to reduce HIV-related stigma [30] and virtual community boards to discuss stigma [31]. OTPs may benefit from implementing similar interventions targeted towards decreasing drug use stigma and rebuilding connections with the larger community, drawing upon existing resources and infrastructure such as CBOs and peer outreach workers.

Family support was identified as a key facilitator of MOUD retention. Consistent with research in other settings [32, 33], the reestablishment of familial relationships following enrollment on MOUD was essential to garnering emotional and instrumental support, which helped to overcome economic barriers to retention, particularly transportation cost which is well documented as a barrier to accessing and remaining on MOUD [12, 34, 35]. However, lack of understanding of methadone and continued mistrust threatened clients’ ability to rebuild trust and maintain family support. As highlighted in our findings and previous research, this lack of knowledge of methadone coupled with stigmatizing attitudes toward people who use drugs can pressure clients to discontinue MOUD [32, 36, 37]. Family-based interventions can complement MOUD and capitalize on the essential role of families in treatment retention, especially in the context of limited social welfare, to support clients to remain on treatment [32, 38]. In this setting, family-based interventions could be useful in mobilizing family members to share the domestic responsibilities with female clients so that these clients may attend clinic and adhere to MOUD. These interventions could correct misinformation that families may hold about methadone, reduce stigmatizing attitudes toward people who use drugs, and rebuild trust, which are all essential for clients to access and maintain familial support.

Clinic policies that restrict access to methadone for clients who break clinic rules were viewed by participants as punitive and as contributing to MOUD attrition. High threshold, low-tolerance MOUD models with strict clinic rules can have negative effects on MOUD adherence and retention [39]. From the health care provider perspective, controlling clinic rules and policies are necessary for patient safety to reduce overdose risks and injection practices that might result in death and blood-borne diseases, such as HIV, and to promote cognitive and behavioral modification. Though some participants in the current study described clinic rules as reinforcing cognitive and affective processes that they believed were necessary for treatment success, most did not. This points to a need to balance patient safety measures and behavioral modification interventions with ensuring that additional barriers to MOUD retention are avoided. Adopting a more client-centered, low threshold treatment approach, in which alternative approaches that do not necessarily involve temporary suspension for clients who violate clinic rules, may help to reduce treatment interruptions, and improve retention. Clinic operating hours, specifically methadone dispensing hours, were also identified by participants as a barrier to MOUD retention. Government efforts currently underway, including the provision of take-away methadone and satellite MOUD dispensaries in targeted neighborhoods, are intended to increase access to methadone and reduce the impacts of common barriers, such as economic and transportation challenges, on MOUD retention. An expansion of clinic methadone dispensing hours at the clinic would further help to increase access for clients, particularly those women clients whose primary source of income is sex work. Furthermore, though the clinic at Muhimbili uses an electronic system to track daily doses of methadone, this system is limited to only tracking doses administered. Clinics would benefit from routinely collecting data on the barriers and facilitators to treatment to better respond to the needs of clients receiving MOUD. These changes at the clinic level would, ultimately, reduce treatment interruption and improve retention over time.

### Study limitations

This study has limitations. Given that this study was limited to a specific OTP clinic in an urban area, the findings may not be generalizable to other settings, including more rural areas. We recruited former clients on MOUD through community outreach workers. Former clients who were reached and agreed to participate in the study may have had different experiences and perceptions compared to clients whom we were unable to reach. Finally, we did not explore provider-patient communications around methadone doses, despite methadone dose being known to affect retention in this setting [10]. This will be an important area for future qualitative research. Despite these limitations, we were able to gain perspectives from a diverse sample of current and former clients, including men and women enrolled in treatment for various time periods.

### Study rigor

We used several measures to ensure the trustworthiness of the results [40]. For credibility, we triangulated findings between current and former clients as well as male and female clients to compare their responses as related to remaining on or discontinuing MOUD. When presenting the results, we relied on direct quotes from participants to illustrate their experiences from their voices. For the transferability of findings, we provided a detailed description of the study setting, specifically the structure, policies, and operations of the MNH OTP clinic, and the demographics of the study sample. For example, for all direct quotes presented, we provided demographic information on clients from whom the quotes were taken. We established dependability by maintaining a decision trail of the analysis and interpretation of findings with memos, coded transcripts, and matrices. For confirmability, the interviews and focus groups were conducted in Swahili, audio recorded, transcribed in Swahili, then translated into English by interviewers who were native Swahili speakers and fluent in English. This process helped to ensure that participants were able to fully express themselves in the language that they felt most comfortable speaking and that the originally meanings of participants’ accounts were maintained. Rich verbatim quotes also added to the confirmability of the study. The study team, many of whom had extensive experience working with clients on MOUD in a research or clinical capacity, also conducted regular debriefing meetings throughout the data collection and analysis periods to discuss interpretations.

### Conclusions

This study highlights several key structural and social factors that are pertinent for MOUD retention, such as economic factors, stigma and discrimination, social support, and clinic policies. Our findings can inform interventions and policies to help clients overcome the economic and social barriers they face while in treatment. Program adjustments at the clinic level are needed and may allow opioid treatment programs offering MOUD to improve retention of clients and ultimately treatment outcomes.

## Data Availability

The datasets generated and/or analyzed during the study are not publicly available to ensure confidentiality of the participants involved but are available from the corresponding author on reasonable request.

## Abbreviations

HIV: Human immunodeficiency virus
MOUD: Medications for opioid use disorder
OTP: Opioid treatment program

## References

[1] Ball JC, Ross A. The effectiveness of methadone maintenance treatment: Patients, programs, services, and outcome. 1991.

[2] Metzger DS, Woody GE, McLellan AT, O'Brien CP, Druley P, Navaline H (1993). Human immunodeficiency virus seroconversion among intravenous drug users in-and out-of-treatment: an 18-month prospective follow-up. J Acquir Immune Defic Syndr.

[3] Sordo L, Barrio G, Bravo MJ, Indave BI, Degenhardt L, Wiessing L (2017). Mortality risk during and after opioid substitution treatment: systematic review and meta-analysis of cohort studies. BMJ.

[4] Umbricht-Schneiter A, Ginn DH, Pabst KM, Bigelow GE (1994). Providing medical care to methadone clinic patients: referral vs on-site care. Am J Public Health.

[5] Fullerton CA, Kim M, Thomas CP, Lyman DR, Montejano LB, Dougherty RH (2014). Medication-assisted treatment with methadone: assessing the evidence. Psychiatr Serv.

[6] Roux P, Carrieri MP, Cohen J, Ravaux I, Poizot-Martin I, Dellamonica P (2009). Retention in opioid substitution treatment: a major predictor of long-term virological success for HIV-infected injection drug users receiving antiretroviral treatment. Clin Infect Dis.

[7] Ubuguyu O, Tran OC, Bruce RD, Masao F, Nyandindi C, Sabuni N (2016). Improvements in health-related quality of life among methadone maintenance clients in Dar es Salaam. Tanzania Int J Drug Policy.

[8] Malta M, Magnanini MM, Strathdee SA, Bastos FI (2010). Adherence to antiretroviral therapy among HIV-infected drug users: a meta-analysis. AIDS Behav.

[9] Lambdin BH, Masao F, Chang O, Kaduri P, Mbwambo J, Magimba A (2014). Methadone treatment for HIV prevention—feasibility, retention, and predictors of attrition in Dar es Salaam, Tanzania: a retrospective cohort study. Clin Infect Dis.

[10] Mlaki DA, Mbwambo JK, McCurdy S, Masao FA, Kaduri PA (2022). Twelve-month treatment retention and associated factors: a comparison of 2 medically assisted therapy clinics in Dar es Salaam. Tanzania. J Addict Med..

[11] Karekla M, Kasinopoulos O, Neto DD, Ebert DD, Van Daele T, Nordgreen T (2019). Best practices and recommendations for digital interventions to improve engagement and adherence in chronic illness sufferers. Eur Psychologist..

[12] Khazaee-Pool M, Moeeni M, Ponnet K, Fallahi A, Jahangiri L, Pashaei T (2018). Perceived barriers to methadone maintenance treatment among Iranian opioid users. Int J Equity Health.

[13] Stark MJ (1992). Dropping out of substance abuse treatment: A clinically oriented review. Clin Psychol Rev.

[14] Najavits LM, Crits-Christoph P, Dierberger A (2000). Clinicians' impact on the quality of substance use disorder treatment. Subst Use Misuse.

[15] Saleem HT, Knight D, Yang C, Kidorf M, Latkin C, Nkya IH (2022). HIV Stigma, HIV status disclosure, and ART adherence in the context of an integrated opioid use disorder and HIV treatment setting in Dar es Salaam. Tanzania. AIDS Care..

[16] Saleem HT, Mushi D, Hassan S, Bruce RD, Cooke A, Mbwambo J (2016). “Can’t you initiate me here?”: Challenges to timely initiation on antiretroviral therapy among methadone clients in Dar es Salaam. Tanzania Int J Drug Policy.

[17] Berkman LF, Glass T, Brissette I, Seeman TE (2000). From social integration to health: Durkheim in the new millennium. Soc Sci Med.

[18] Charmaz K (2006). Constructing grounded theory: A practical guide through qualitative analysis.

[19] Hser Y-I, Polinsky ML, Maglione M, Anglin MD (1999). Matching clients’ needs with drug treatment services. J Subst Abuse Treat.

[20] Magura S, Marshall T (2020). The effectiveness of interventions intended to improve employment outcomes for persons with substance use disorder: an updated systematic review. Subst Use Misuse.

[21] Platt JJ (1995). Vocational rehabilitation of drug abusers. Psychol Bull.

[22] Harrison J, Krieger MJ, Johnson HA (2020). Review of individual placement and support employment intervention for persons with substance use disorder. Subst Use Misuse.

[23] Kidorf M, Hollander JR, King VL, Brooner RK (1998). Increasing employment of opioid dependent outpatients: an intensive behavioral intervention. Drug Alcohol Depend.

[24] Room R (2005). Stigma, social inequality and alcohol and drug use. Drug Alcohol Rev.

[25] Semple SJ, Grant I, Patterson TL (2005). Utilization of drug treatment programs by methamphetamine users: The role of social stigma. American Journal on Addictions.

[26] Ahern J, Stuber J, Galea S (2007). Stigma, discrimination and the health of illicit drug users. Drug Alcohol Depend.

[27] Livingston JD, Milne T, Fang ML, Amari E (2012). The effectiveness of interventions for reducing stigma related to substance use disorders: a systematic review. Addiction.

[28] Luty J, Rao H, Arokiadass SMR, Easow JM, Sarkhel A (2008). The repentant sinner: methods to reduce stigmatised attitudes towards mental illness. Psychiatr Bull.

[29] Luty J, Umoh O, Nuamah F (2009). Effect of brief motivational interviewing on stigmatised attitudes towards mental illness. Psychiatr Bull.

[30] Kerr JC, Valois RF, DiClemente RJ, Carey MP, Stanton B, Romer D (2015). The effects of a mass media HIV-risk reduction strategy on HIV-related stigma and knowledge among African American adolescents. AIDS Patient Care STDS.

[31] Flickinger TE, DeBolt C, Xie A, Kosmacki A, Grabowski M, Waldman AL (2018). Addressing stigma through a virtual community for people living with HIV: a mixed methods study of the positivelinks mobile health intervention. AIDS Behav.

[32] Kermode M, Choudhurimayum RS, Rajkumar LS, Haregu T, Armstrong G (2020). Retention and outcomes for clients attending a methadone clinic in a resource-constrained setting: a mixed methods prospective cohort study in Imphal. Northeast India Harm Reduction J.

[33] Silva TC, Andersson FB (2021). The “black box” of treatment: Patients’ perspective on what works in opioid maintenance treatment for opioid dependence. Substance Abuse Treatment Prev Policy.

[34] Abadie R, McLean K, Habecker P, Dombrowski K (2021). Treatment trajectories and barriers in opioid agonist therapy for people who inject drugs in rural Puerto Rico. J Subst Abuse Treat.

[35] Hayashi K, Ti L, Ayutthaya PPN, Suwannawong P, Kaplan K, Small W (2017). Barriers to retention in methadone maintenance therapy among people who inject drugs in Bangkok, Thailand: a mixed-methods study. Harm Reduct J.

[36] Larney S, Zador D, Sindicich N, Dolan K (2017). A qualitative study of reasons for seeking and ceasing opioid substitution treatment in prisons in New South Wales. Australia Drug Alcohol Rev.

[37] Woo J, Bhalerao A, Bawor M, Bhatt M, Dennis B, Mouravska N (2017). “Don’t judge a book by its cover”: A qualitative study of methadone patients’ experiences of stigma. Subst Abuse.

[38] Feng N, Lin C, Hsieh J, Rou K, Li L (2018). Family related factors and concurrent heroin use in methadone maintenance treatment in China. Subst Use Misuse.

[39] McElrath K (2018). Medication-assisted treatment for opioid addiction in the United States: Critique and commentary. Subst Use Misuse.

[40] Elo S, Kääriäinen M, Kanste O, Pölkki T, Utriainen K, Kyngäs H (2014). Qualitative content analysis: a focus on trustworthiness. SAGE Open.

